# The Common Accidents of Every-Day Life

**Published:** 1887-10-29

**Authors:** Robert A. P. Forrester


### THE COMMON ACCIDENTS OF EVERY-DAY LIFE.

By Robert A. P. Forrester, B.A.Dub., M.B. and C.M.Edin.

### INFANCY.

"First the infant, mewling and puking in his nurse's arms."

(*Continued from p. 12.*)

*Death.*—Asphyxia or suffocation is not uncommon among children, especially among the poorer classes. One may often read in the newspapers of children having been *overlaid*, as it is called; but in very many instances no marks of pressure have been discovered, and on *post-mortem* examination the right cavities of the heart have been found gorged with blood, and the general surface of the body is livid, clearly showing death by asphyxia.

The true cause of death is the inhalation of the contaminated air inspired, which has slowly accumulated under the bed-clothes, drawn over the child's head to keep it warm. A mother or careless nurse may have gone to sleep while allowing the child to continue sucking. The child, after a time, loses the nipple, and buries its head in the bed-clothes. The mother awakes in the morning to find a corpse by her side. Should, however, any signs of life be apparent, the best method to pursue is to expose the body to cold air, dash cold water over the face and chest, and set up artificial respiration, if necessary. Pye-Chavasse has given in one of his works some excellent rules for the prevention of this accident:—"1. Let the baby when asleep have plenty of room in the bed. 2. Do not allow him to be too near you. 3. Let him lie fairly on his side or his back. 4. See that his mouth be not covered by the bed-clothes. 5. Never let him lie low in the bed. 6. Let there be no pillow near the one his head is resting on, lest he roll to it, and bury his head in it."

*Cut Finger.*—If a child has cut its finger, merely tie a rag round it, after having cleansed the wound of any dirt that may adhere. If the part bleed very much, sponge the wound with cold water, and exert a little gentle pressure.

*A Fall.*—A nurse may let a child fall and injure it, perhaps in the back, or it may sustain a fracture of a limb or other serious injury. The best plan is at once to send for a medical man, "home aid" in such cases not being sufficient. A gentleman of my acquaintance, a tall, strong, handsome man, was dropped by his nurse when an infant, and sustained a severe fracture at the ankle-joint. The nurse was afraid of the consequences of her carelessness, and omitted to inform her mistress of the circumstance, with the result that the injury was not found out for months afterwards. He was a cripple for life; and although all that surgery could do for him was done, he was never able to put his foot flatly on the ground.

*Swallowing Lotions, etc.*—If a child has accidentally swallowed a liniment or lotion containing opium, send immediately for a medical man. In the meantime, give him a mustard emetic (a tablespoonful of mustard in half-a-pint of warm water, or half that quantity if the child be very young), and let him continue to drink warm water until free vomiting is produced. Throw cold water over his face and head. Rouse him by walking him about and slapping him on the back, pinching him, and shouting at him. If you allow him to go to sleep he will probably sleep to wake no more.

*Foreign Bodies in the Ear.*—Children often insert peas, beans, beads, etc., into the ear, with more or less injury to the organ. In such a case, first of all ascertain that a foreign body is in the canal, and if so, do not attempt to extract with forceps, unless you can easily lay hold of it. Attempts of this kind often result in pushing the article further in. The best plan is to syringe the ear with warm water; use a large brass syringe; pull the pinna upwards to straighten the canal, incline the head of the patient towards the basin, and put the point of the syringe in the upper surface of the canal. In this way the bead or pebble will easily be washed out by the current of water injected. If it cannot be syringed out, it will probably not be extracted by the use of instruments. It may be necessary to give the child chloroform before the operation. All operations, however slight, should be performed by a medical man only.

(*To be continued.*)

